# Integrative Analysis of Exosomal miR-452 and miR-4713 Downregulating *NPY1R* for the Prevention of Childhood Obesity

**DOI:** 10.1155/2022/2843353

**Published:** 2022-03-30

**Authors:** Xiaoyan Feng, Ye Ding, Min Zhou, Na Song, Yanhong Ding

**Affiliations:** ^1^Department of Nursing, The Affiliated Wuxi Children's Hospital of Nanjing Medical University, Jiangsu Province 214000, China; ^2^Department of Endocrinology, The Affiliated Wuxi Children's Hospital of Nanjing Medical University, Jiangsu Province 214000, China; ^3^Department of Orthopedics and Neurosurgery, The Affiliated Wuxi Children's Hospital of Nanjing Medical University, Jiangsu Province 214000, China

## Abstract

Neuropeptides are associated with childhood obesity and exploring their regulatory mechanisms may reveal new insights for novel treatments. Childhood obesity data were downloaded from the GEO database and were used to screen for differentially expressed neuropeptides in patients with obesity. NPY1R expression was significantly upregulated in children with obesity compared to children without obesity (*p* < 0.05). The GEO database was used to filter differentially expressed miRNAs in patients with obesity. And hsa-mir-4713 and hsa-mir-452 were found significantly downregulated in adipose tissue. The GEO, TRRUST, and TFacts databases were used to screen all transcription factors for differentially expressed genes (DEGs). The potential regulatory networks between the differentially expressed miRNAs, TFs, and neuropeptides were mapped. In the constructed NPY1R regulatory network, the transcription factors TCF4, HEY1, and GATA3 are significantly associated with *NPY1R*. *TCF4* and *HEY1* were positively correlated with *NPY1R*, while GATA3 was negatively correlated with NPY1R. In the clinical peripheral blood samples, NPY1R, TCF4, and HEY1 were significantly more expressed in the obesity and the obesity with fracture group compared to the control group, while there was no statistically significant difference between the obesity group and the obesity with fracture group in terms of expression. The expression of GATA3, miR-452, and miR-4713 was also significantly lower in the obesity and the obesity with fracture groups when compared to the NC group. Therefore, NPY1R, TCF4, HEY1, GATA3, miR-452, and miR-4713 may be risk factors for fracture in obese children. The potential NPY1R regulatory function was exerted by two pathways: positive regulation caused by TCF4 and HEY1 acting on miR-4713 and negative regulation via GATA3 acting on miR-452. Potential NPY1R-related targets for the treatment of childhood obesity were provided in this study.

## 1. Background

According to a recent report by the World Health Organization (WHO), approximately 41 million children aged 5 and under were overweight or obese worldwide in 2016, and 18% of young people aged 5 to 19 were overweight or obese [1]. Childhood obesity has been on the rise worldwide over the past three decades [2]. Being overweight or obese is one of the risk factors of premature death. When childhood obesity persists into adulthood, the risk of developing chronic diseases increases significantly [3]. There is growing evidence that obesity affects bone health in children, as well as the typical complications of obesity in adults, including hyperlipidemia, hypercholesterolemia, hypertension, metabolic syndrome, nonalcoholic fatty liver disease, and type 2 diabetes [3–6]. Obesity increases the risk of fractures [7]. Adipocytes and osteoblasts originate together from mesenchymal stem cells (MSC) in the bone marrow [8]. There is an inverse relationship between increased adipocytes in the bone marrow and bone formation [1]. Increased adipocytes in the bone marrow can affect osteoblast differentiation and function, increase osteoclast activity, and hinder bone mineralization [9]. Romagnoli et al. showed that the accumulation of abdominal fat has a negative effect on the microstructure of bone trabeculae [10]. Adipocytes have an endocrine function and can also affect bone through the endocrine pathway, leading to reduced bone mass and even osteoporosis. For example, leptin inhibits osteoprotegerin and osteoclast nuclear factor receptor activator ligands at higher concentrations, causing a decrease in bone mineral density (BMD), which leads to susceptibility to fragility fractures [11].

Moreover, childhood obesity has become a global public health problem that affects not only the physical and mental health of children but also their health and quality of life in adulthood. Childhood overweight and obesity lead to increased behavioral health problems in children and can affect children and adolescents in the long term [12]. Therefore, it is urgent to develop a feasible program to prevent and treat childhood obesity as soon as possible. One potential avenue of treatment would be the regulation of neuropeptides. Neuropeptides are signaling molecules in the central nervous system that have been shown to regulate appetite. Neuropeptide Y (NPY) is widely distributed in the central and peripheral nerves and is an important neurotransmitter and a proappetite factor [13]. NPY acts through its receptors to promote feeding, energy storage, and adipose tissue accumulation [13–15]. NPY can thus contribute to obesity by promoting the accumulation of white adipose tissue (WAT) [16]. In addition, NPY inhibits the thermogenic activity of the body's brown adipose tissue (BAT), thereby decreasing the body's metabolic rate and reducing energy expenditure [17].

Numerous studies in recent years have found that transcription of neuropeptides is regulated by miRNAs [18, 19]. Recent studies have shown that miRNAs are involved in various biological processes related to obesity, including adipocyte differentiation, lipid metabolism, and changes in insulin sensitivity [20–22]. It has been reported that the miRNA expression profiles of obese patients and those with normal Body Mass Index (BMI) differ significantly [23]. miR-143 was the first miRNA reported to be associated with the development of adipocyte differentiation. Overexpression of miR-143 promotes adipocyte formation, suggesting that it is effective in promoting WAT production [24]. The expression of miR-455 increased significantly with the formation of brown fat cells, suggesting that miR-455 is involved in energy depletion [25]. Sun et al. identified miRNA-193b-365 as a key factor in the regulation of brown fat differentiation [26]. miR-122 is involved in hepatic lipid metabolism that triggers obesity [27]. A prospective study of up to 810 people found that high miR-122 levels were positively associated with obesity [28]. MicroRNAs may therefore be involved in the pathogenesis of childhood obesity through the regulation of neuropeptides or their associated transcription factors.

This study is aimed at revealing the neuropeptides associated with pediatric obesity, fractures, and their regulatory network by performing database analyses and collecting clinical peripheral blood samples for validation.

## 2. Methods

### 2.1. Patients

Peripheral blood samples were collected from 10 obese patients with distal radius fracture and 10 obese patients without fractures, all females aged 5-11 years attending Wuxi Children's Hospital of Nanjing Medical University from 2015 to 2021. The inclusion criteria for children with fractures were as follows: (i) a clear history of trauma, no combined vascular or neurological injuries, and a confirmed radial diaphysis fracture on X-ray; (ii) confirmed obesity; (iii) age < 16 years; and (iv) complete clinical and imaging data. The following are the exclusion criteria: (i) pathological fractures, osteogenesis imperfecta, and other bone metabolic diseases; (ii) combination of other systemic pathologies that predispose to fracture or affect the healing process; (iii) recurrent fractures; (iv) congenital ulnar radial deformity or other neuromuscular bone dysfunction; (v) multiple fractures or combination of other systemic injuries; and (vi) incomplete clinical and imaging data and failure to follow up. Obesity was determined by taking height and weight data and calculating Body Mass Index (BMI) according to the following formula: BMI (kg/m^2^) = weight (kg)/[height (m)] [2]. Patients with BMI ≥ 30 were considered obese, following WHO obesity classification guidelines. Peripheral blood samples were taken randomly from 10 children without fractures from the normal physical examination population. According to the WHO guidelines, all patients in this normal group were female, aged 5-10 years, and had normal BMI (between 18.5 and 25). The inclusion of the study population was in accordance with the principle of randomisation and double blinding. All three groups were free of other diseases and had not received any treatment or medication 3 months prior to blood sampling. There were no statistical differences in clinical sample characteristics between the three groups except for BMI. Informed consent was obtained from all patients and their guardians after the purpose of the study was explained. The Ethics Committee of the Wuxi Children's Hospital of Nanjing Medical University approved the study.

### 2.2. Chip Data Selection and Variance Analysis

Childhood obesity-related datasets were searched in the GEO GeneChip public database in NCBI (http://www.ncbi.nlm.nih.gov/geo/). A gene expression data set of 27 samples, GSE9624, was selected for analysis. Data were collected from adipose tissue samples taken from 14 obese and 13 nonobese children who underwent appendectomy [29]. The GSE50574 and GSE68885 datasets contained information on adipocyte-derived exosomes from obese and normal populations [30]. Therefore, the GSE50574 and GSE68885 datasets were used to screen for differentially expressed exosomal miRNAs in obese patients. The filter criteria used were *p* value < 0.05 and absolute log-fold change ∣logFC | ≥131 [31, 32].

### 2.3. Network Construction of TF-miRNA, miRNA-NPY1R, and TF-NPY1R

miRNAs that bind to NPY targets were predicted using miRwalk2.0. miRwalk 2.0 is a dataset for identifying potential regulatory targets of miRNAs based on computer prediction. It integrates 12 online databases to perform this analysis [33]. Therefore, miRwalk2.0 was used to construct potential miRNA-NPY1R relationship pairs. TransmiR v2.0 is an efficient database for the study of regulatory miRNA transcription factors, containing comprehensive information on TF-miRNA regulation, showing the TF-miRNA regulatory rules for each TF and miRNA or a specific disease [34, 35]. TransmiR v2.0 was used to construct potential TF-miRNA relationship pairs. The JASPAR database is an open-source, public database containing information on transcription factors and DNA binding sites and has been used to analyze potential transcription factors (TFs) regulating Neuropeptide Y Receptor Y1 (NPY1R) [36]. TRRUST (Transcriptional Regulatory Relationships Unraveled by Sentence-based Text mining) is a database of transcriptional regulatory relationships, including the target genes of transcription factors and the regulatory relationships between transcription factors [35]. Furthermore, OncoBinder is a tool for evaluating proteomic interaction data and was used to identify proteins that potentially interact with NPR1R [37].

### 2.4. Enrichment Analysis

As per previous research, enrichment analysis was performed with R software (version 3.6.0) [38–40]. The three enrichment analyses Gene oncology (GO), Kyoto Encyclopedia of Genes and Genomes (KEGG), Gene Set Enrichment Analysis (GSEA) were performed. The clusterProfiler package (version 3.14.3) was used for enrichment analysis, and the http://org.hs.eg/.db package (version 3.10.0) was used for gene annotation. A false discovery rate (FDR) < 0.25 and an adjusted *p* value (*p*.adjust) < 0.05 was defined as significant enrichment.

### 2.5. Quantitative PCR Assays

Detection of miRNA expression levels was performed using a reverse transcription kit (Bioteke) according to the manufacturer's instructions. Briefly, 20 *μ*L reaction mixtures containing tissue samples were incubated at 42°C for 60 min and 80°C for 5 min, then reverse-transcribed into cDNA and stored at -20°C in the refrigerator. The reaction was predenatured at 95°C for 2 min and 40 cycles at 95°C for 15 s and 60°C for 1 min. The mRNA expression levels were measured by extracting total RNA from blood using Trizol. Synthesis of cDNA was then performed according to the instructions detailed above. The primers were then mixed with cDNA and SYBR Green dye for real-time quantitative PCR. Expression was calculated based on the results of three independent experiments using the 2-*ΔΔ*Ct method. The PCR parameters were 2 min at 95°C, 1 cycle at 95°C for 5 s, and 30 s at 60°C. Cycle threshold (Ct) values for the PCR reactions were obtained with a 7500 real-time quantitative fluorescence PCR instrument (ABI, USA).

### 2.6. Statistical Analysis and Visualization

All experiments were repeated at least three times. The data were expressed as the mean ± standard deviation, and the results were analyzed using R software. Differences were considered significant at *p* < 0.05. Sankey diagrams were drawn to visualize the interrelationships between molecular markers.

## 3. Results

### 3.1. Characterization of NPY1R Expression and Function in Children with Obesity

**Figure 1:**
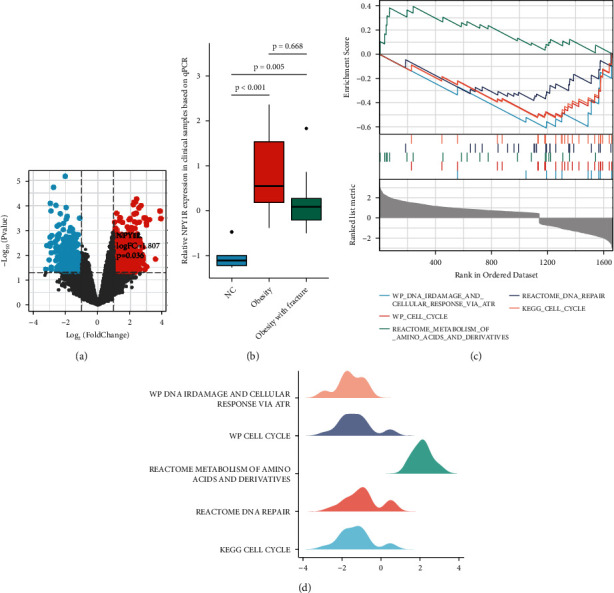
Characterization of NPY1R expression and function in children with obesity. The result of analysis of variance tests on the GSE9624 dataset (a); expression of NPY1R mRNA in the normal control, obesity, and obesity with fracture groups by qPCR (b); visualization of NPY1R-related GSEA (c, d).

The results of the analysis of variance (ANOVA) test in the dataset GSE9624 showed that NPY1R expression was upregulated in obese children and was statistically significant (*p* = 0.036) (Figure 1(a)). Next, qPCR was used to detect the expression of NPY1R mRNA in the three groups (Figure 1(b)). Consistent with the results of the differential analysis of the dataset, the qPCR results of the clinical samples showed that NPY1R was significantly more highly expressed in the obesity and obesity with fracture groups relative to the NC group, while there was no statistically significant difference between the obesity and obesity with fracture groups. In order to further understand the primary role of NPY1R in humans, a GSEA enrichment analysis was performed (Figures 1(c) and 1(d)). GSEA results suggest that NPY1R was associated with DNA infrared damage and cellular response via ATR, cell cycle, reactome metabolism of amino acids and derivatives, reactome DNA repair, and cell cycle signaling pathways.

### 3.2. Potential miRNAs and Transcription Factors Regulating NPY1R

**Figure 2:**
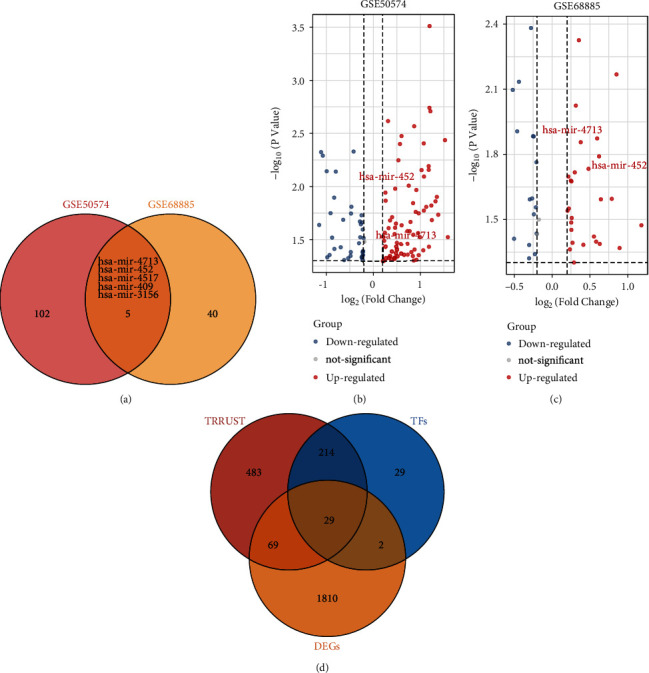
Potential miRNAs and transcription factors regulating NPY1R. Venn diagram demonstrating common differential miRNAs in the GSE50574 and GSE68885 datasets (a); volcano map showing that hsa-mir-452 and mir-4713 were found to be upregulated in normal tissues relative to adipose tissue expression in GSE50574 and GSE68885 (b, c); transcription factors associated with differentially expressed genes (DEGs) in adipose tissue of obese patients revealed with Venn diagrams (d).

Comparing the GSE50574 and GSE68885 datasets revealed 102 and 40 differentially expressed miRNAs in obese and lean adipocyte-derived exosomes, respectively. There are five differential miRNAs in the intersection of the two datasets: hsa-mir-4713, hsa-mir-452, hsa-mir-4517, hsa-mir-409, and hsa-mir-3156 (Figure 2(a), Table 1). The miRwalk2.0 database was used to predict the interaction of these miRNAs with NPY1R. The results showed that hsa-mir-4713 and hsa-mir-452 interacted with NPY1R. Plotting the results on a volcano map showed that hsa-mir-4713 and hsa-mir-452 were significantly downregulated in adipose tissue relative to normal tissue (Figures 2(b) and 2(c)). Transcription factors associated with differentially expressed genes (DEGs) in adipose tissue of obese patients revealed with Venn diagrams (Figure 2(d)).

### 3.3. Construction of a Potential Regulatory Network for NPY1R

**Figure 3:**
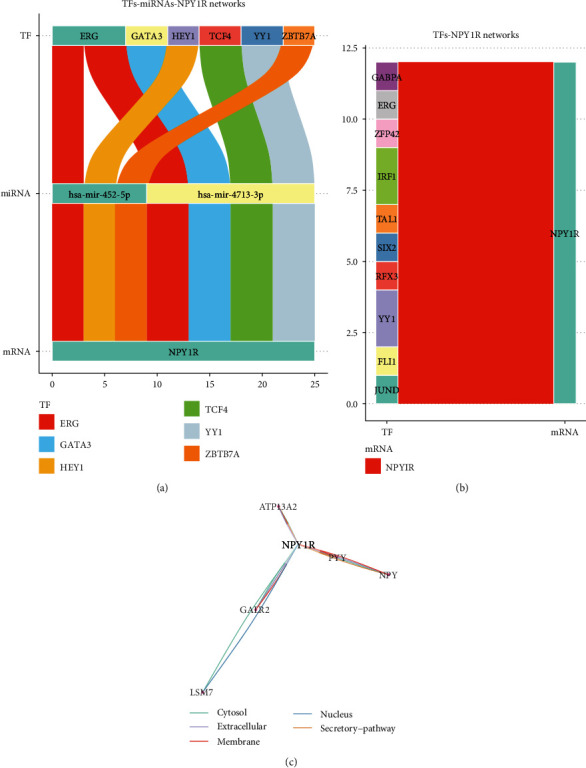
Construction of a potential regulatory network for NPY1R. Sankey diagram demonstrating a series of TFs-miRNAs-NPY1R regulatory networks (a); Sankey diagram showing potential TFs regulating NPY1R (b); graphs showing potential functionally relevant interaction factors and their scores, with all connecting lines indicating statistical significance (*p* < 0.05) (c).

The TransmiR database was used to search and predict TF-miRNA regulatory relationships. The JASPA and OncoBinder databases were used to predict potential regulatory TFs for NPY1R. Sankey diagrams showing a series of TFs-miRNAs-NPY1R regulatory networks (Figure 3(a)) and TFs that may regulate NPY1R (Figure 3(b)) were constructed based on the findings. In addition, the interaction factors of NPY1R were also analyzed using the OncoBinder model to identify functionally relevant interactions. The scores of the candidate interactors were plotted in a graph, with all lines indicating statistical significance (*p* < 0.05; Figure 3(c)).

### 3.4. Enrichment Analysis of NPY1R

**Figure 4:**
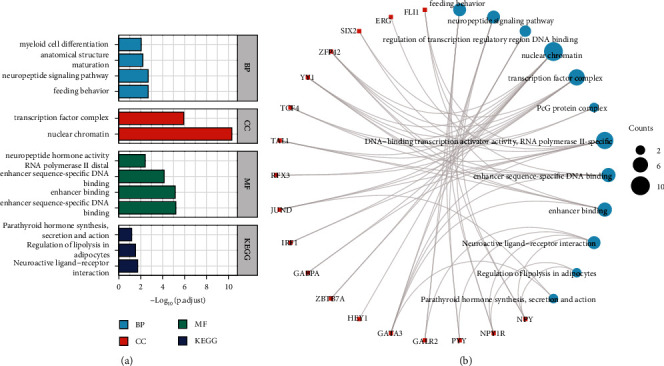
Enrichment analysis of *NPY1R*. GO and KEGG enrichment analysis of *NPY1R* (a); the enrichment networks of *NPY1R* are also shown (b).

To complete the regulatory network, GO and KEGG enrichment analysis was performed in potential NPY1R-interacting proteins. GO and KEGG enrichment analysis showed that the functions of NPY1R are mainly related to myeloid cell differentiation, anatomical structure, maturation, neuropeptide signaling pathway, feeding behavior, transcription factor complex, nuclear chromatin, neuropeptide hormone activity, RNA polymerase II distal enhancer sequence-specific DNA binding, enhancer binding, and enhancer sequence-specific DNA binding; parathyroid hormone synthesis, secretion, and action; and regulation of lipolysis in adipocytes and neuroactive ligand-receptor interaction (Figure 4(a)). The regulatory network between the differentially expressed miRNAs, TFs, and NPY1R, as well as the enriched signaling pathway, is shown in Figure 4(b).

### 3.5. Screening for Core Transcription Factors Regulating NPY1R and Expression Analysis of Clinical Samples

**Figure 5:**
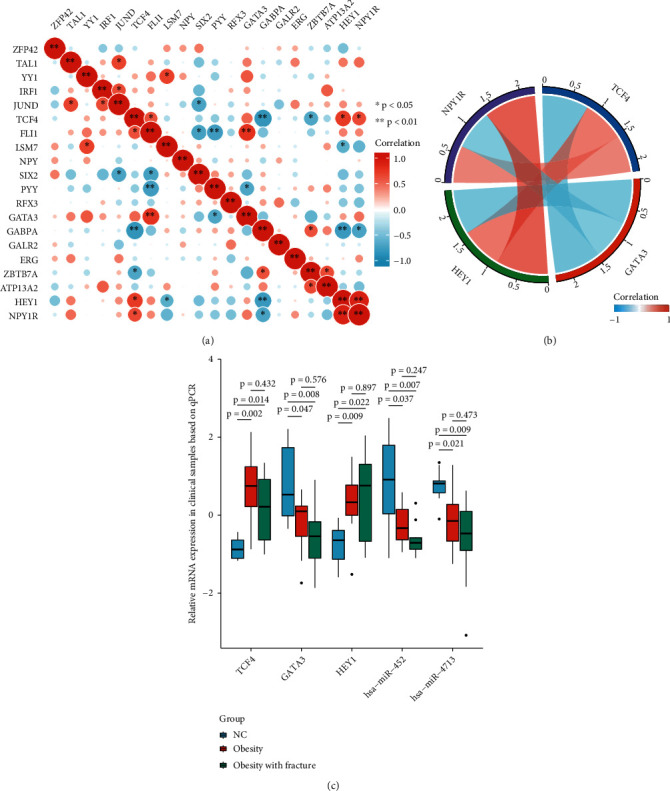
Screening for core transcription factors regulating NPY1R and expression analysis of clinical samples. Correlation analysis results on the identified regulators (a); Pearson's correlation (NPY1R, TCF4, HEY1, and GATA3) analysis between transcription factors (b); relative mRNA levels of TCF4, GATA3, HEY1, miR-452, and miR-4713 in clinical samples (c).

**Figure 6:**
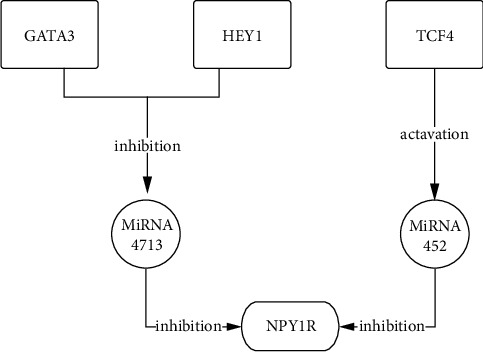
Mechanistic map of the TF-miRNA-NPY1R regulatory network demonstrates two signaling pathways associated with NPY1R in childhood obesity.

Next, all the regulators in the network in Figure 4 were subjected to correlation analysis. The results showed that TCF4, HEY1, GATA3, and NPY1R were significantly correlated. While TCF4, HEY1, and NPY1R were positively correlated, GATA3 was negatively correlated with NPY1R (Figures 5(a) and 5(b)). The mRNAs for the TFs TCF4, GATA3, and HEY1, as well as miR-452- and miR-4713-related mRNAs, were detected using qPCR in the clinical peripheral blood samples, and the results were consistent with those obtained by our earlier bioinformatics analysis (Figure 5(c)). Mechanistic map of the TF-miRNA-NPY1R regulatory network demonstrates two signaling pathways associated with NPY1R in childhood obesity (Figure 6).

## 4. Discussion

In this study, we used bioinformatics analyses to investigate the neuropeptides associated with childhood obesity and their regulatory mechanisms and collected clinical peripheral blood samples for validation. The construction and study of the regulatory network between differentially-expressed miRNAs, TFs, and NPY1R and the enriched signaling pathways will further reveal the pathogenesis of childhood obesity and guide the clinical management of childhood obesity and related injuries such as fractures. Here, we briefly went through the major members of the network and attempted to elucidate their possible roles.

NPY, a 36-amino acid peptide, is commonly expressed in the human central and peripheral nervous system and is involved in various physiological processes, including the maintenance of bone homeostasis, energy synthesis, and the feeding reflex [41]. Meanwhile, the NPY receptors are a family of protein-coupled receptors, including Y1R, Y2R, Y4R, Y5R, and Y6R [42]. The receptor NPY1R is also known as the appetite-promoting receptor. It has been shown that NPY1R mRNA expression is increased in diet-induced obesity-sensitive rats and decreased in diet-induced resistant rats, suggesting a correlation between Y1R gene expression levels and a genetic predisposition to the development of obesity [43]. Rapid food deprivation experiments also show a significant reduction in NPY1R expression in the hypothalamic regions [44]. In line with previous studies, we found that NPY1R expression was significantly more highly expressed in obese children—the obesity and obesity with fracture groups—than in nonobese children. Obesity is an expansion of adipose tissue with chronic low-level systemic inflammation, which leads to the accumulation of ectopic fat cells in the bone marrow cavity. This may impair bone regeneration and lead to osteoporosis, in turn increasing the susceptibility to fragility fractures [45]. In addition, obese children are at significantly higher risk of developing type 2 diabetes, coronary heart disease, chronic kidney disease, and cancer [12, 46, 47]. Therefore, the active search for molecular mechanisms related to childhood obesity is of great clinical importance for preventing and treating childhood obesity and the resulting fractures. Previous studies have found that NPY also plays an important role in the regulation of bone metabolism [48]. NPY binding to Y1R is directly involved in the differentiation of bone progenitor cells and the regulation of osteoblast activity [49] and the differentiation of mesenchymal stem cells into osteoblasts and bone synthesis in general [50]. Under stress, NPY in the blood acts directly on the Y1R of osteoblasts to inhibit osteoblast activity [51]. In contrast to the results of previous studies, our study found no statistically significant difference in NPY1R between the children in the obesity and the obesity with fracture groups. However, this may be related to the small sample size and needs to be further validated in the future. Despite the small sample size, we successfully validated the network we constructed around NPY1R. This model shows three TFs (TCF4, HEY1, and GATA3) and two miRNAs (miR-452 and miR-4713) strongly correlated to its expression and possible function.

HEY1 is a member of the basic-helix-loop-helix (bHLH) transcription factor superfamily HES/HEY, which are known to play important roles in cell growth, proliferation, differentiation, and apoptosis [52]. HEY1 is also a common target of several tumorigenesis-related signaling pathways such as Notch, TGF-*β*/BMP, and Smad [53–55]. Previous studies on HEY1 in obesity are scarce, however. It has been shown that Notch and its target gene, the HES/HEY family, are activated in patients with obesity-related liver disease and may represent a therapeutic target for patients with obesity-related liver disease [56]. Here, HEY1 was identified for the first time as a core transcription factor associated with NPY1R expression and significantly correlated with high expression of NPY1R in obese patients. This result was confirmed using clinical peripheral blood samples. HEY1 expression levels were significantly elevated in children with obesity than in those who did not have obesity, and there was no significant difference between the obesity and obesity with fracture groups.

TCF4 (T-cell factor 4), a transcription factor with an HMG-box, is also known as transcription factor E2-2 or TCF7L2 [57, 58]. TCF4 interacts with *β*-catenin to form a transcriptional regulatory complex, which is involved in regulating the Wnt signaling pathway and downstream-related gene expression [59]. Wnt/*β*-catenin is a key negative regulatory signaling pathway for adipogenesis. In the Wnt pathway, stable *β*-catenin enters the nucleus and binds to the TCF/LEF (lymphoid enhancer factor) family of transcription factors, which inhibit the expression of adipogenic transcription factors [60]. In addition, the Wnt/*β*-catenin signaling pathway has important implications for bone regeneration and skeletal development [61]. Both *β*-catenin and TCF proteins regulate the expression of osteoprotegerin, which is present in osteoblasts and suppresses osteoclast differentiation [62]. Under normal conditions, *β*-catenin in the classical Wnt signaling pathway binds to downstream TCF4/TCF7L2 molecules and promotes bone formation [63, 64]. Our study was the first to identify TCF4 as a core transcription factor associated with NPY1R expression and show that it is significantly and positively correlated with NPY1R. This result was also confirmed in clinical peripheral blood samples. Compared with the NC group, TCF4 was significantly more expressed in the obesity group and the obesity with the fracture group. However, there was no statistically significant difference between the obesity group and the obesity with fracture group.

GATA3 (GATA-binding protein 3) is a member of the zinc-finger protein transcription factor family and regulates gene expression in multiple tissues and organs during embryogenesis, including hematopoietic cells, the skin, kidney, mammary glands, and the central nervous system [65–67]. GATA3 is mainly found in the preadipocytes of white adipose tissue and is a marker molecule of preadipocytes. It has been shown that GATA3 can inhibit adipocyte lipogenesis by suppressing PPAR-*α* promoter activity. This causes cells to remain in the preadipocyte stage [68]. Similar to previous findings, our study found that GATA3 was a core transcription factor associated with NPY1R expression and was significantly negatively associated with high NPY1R expression in patients with obesity. In our clinical samples, the expression of GATA3 was significantly lower in the obesity group and the obesity with fracture group compared to the NC group.

MicroRNAs are a class of noncoding RNAs of approximately 22 nucleotides in length, which have a variety of functions such as regulating growth and development, apoptosis, cell proliferation, and hematopoietic processes [69–72]. Previous studies have shown that adipocytes and macrophages in adipose tissue can influence the expression of target proteins and regulate the function of target cells through miRNAs, adipokines, inflammatory mediators, and other cellular factors, regulating lipid metabolism and the intracellular environment [73]. Circulating levels of miRNAs also correlated strongly with the degree of obesity and its complications [74].

There are few studies on the association of miR-4713 and miR-452 with obesity and fractures. It has been found that silencing miR-452 in mice or cultured adipocytes increased lipid uptake in white fat but reduced glucose uptake and mitochondrial respiration in brown fat. Differential expression of miR-452 gene has similarly been associated with adipogenesis, mitochondrial metabolism, and glucose uptake in white and brown adipose tissue [75]. We discovered here for the first time, through bioinformatics analyses, that hsa-mir-4713 and hsa-mir-452 were differentially expressed in obese and normal human adipocyte-derived exosomes and interacted with NPY1R. The miRNAs hsa-mir-4713 and hsa-mir-452 were significantly downregulated in adipose tissue compared to normal tissue. Furthermore, when compared to the normal group in our clinical validation, hsa-mir-4713 and hsa-mir-452 were significantly lower in the obese and the obese with fracture groups, but there was no statistically significant difference between these two groups. It can thus be hypothesized that the expression of hsa-mir-4713 and hsa-mir-452 was associated with obesity rather than fractures.

Based on these results, we identified two novel NPY1R-associated signaling pathways in childhood obesity. The first pathway involves the transcription factors HEY1 and TCF4, which are positively correlated with NPY1R and are highly expressed in obese patients. Both act on microRNA 4713, causing the downregulation of microRNA 4713 expression and the upregulation of NPY1R expression. The second pathway involves GATA3, which has reduced expression in obese patients, resulting in the downregulation of miR-452 and the upregulation of NPY1R expression. These two pathways might be further investigated to yield new therapies for children with obesity.

There are some limitations to this study. The clinical part of this study was a retrospective study, comprising only female subjects from a narrow age range. The number of cases was also small, and there was selective bias. As our findings were based on bioinformatics methods and validated in the clinical samples, a larger sample size and age range would be essential to ensure the validity of our findings. Additionally, the mechanisms of each signaling pathway still need to be further explored in animal models.

## 5. Conclusion

Based on a bioinformatics approach, we discovered two TF-miRNA-NPY1R pathways involved in the development of childhood obesity but were not associated with fractures in obese children. We predicted the potential mechanisms of miR-452 and miR-4713 in the regulation of NPY1R in childhood obesity, providing a new avenue for potential treatments in this disease. These treatments may then assist in early clinical intervention and individualized treatment planning.

## Figures and Tables

**Table 1:** Exosomal miRNAs potentially regulating NPY1R expression.

miRNA	Target	Start	End	Binding	Energy	Position
hsa-miR-4713-3p	NPY1R	1061	1100	1	-23.2	CDS
hsa-miR-4713-3p	NPY1R	38	56	0.846153846	-19.6	5UTR
hsa-miR-452-5p	NPY1R	1612	1635	0.961538462	-21	3UTR
hsa-miR-452-5p	NPY1R	2202	2215	0.846153846	-16.3	3UTR

## Data Availability

Childhood obesity-related datasets are searched in the GEO GeneChip public database in NCBI, including GSE9624, GSE50574, and GSE68885. Clinical data involved in the study can be obtained by contacting the corresponding author.

## Abbreviations

BAT: Brown adipose tissue
BMD: Bone mineral density
BMI: Body Mass Index
GATA3: GATA-binding protein 3
miRNA: MicroRNA
MSC: Mesenchymal stem cells
NPY: Neuropeptide Y
TCF4: T-cell factor 4
WAT: White adipose tissue
DEGs: Differentially expressed genes
TFs: Transcription factors.
